# Early recovery in the first 24 months of treatment in first-episode schizophrenia-spectrum disorders

**DOI:** 10.1038/s41537-019-0091-y

**Published:** 2020-01-08

**Authors:** Lebogang Phahladira, Hilmar K. Luckhoff, Laila Asmal, Sanja Kilian, Frederika Scheffler, Stefan du Plessis, Bonginkosi Chiliza, Robin Emsley

**Affiliations:** 1 Department of Psychiatry, Stellenbosch University, Stellenbosch, South Africa; 2 Department of Psychiatry, Nelson R Mandela School of Medicine, University of Kwazulu-Natal, Kwazulu-Natal, South Africa

**Keywords:** Schizophrenia, Psychosis

## Abstract

Studies assessing the treatment outcomes in first-episode schizophrenia have reported mixed results. While symptom improvement is frequently robust, when other domains are considered outcomes are generally poorer. We explored response trajectories, rates and predictors of recovery in the domains of core psychopathology, clinician-rated social and occupational functioning and patient-rated quality of life over 24 months of treatment in 98 patients with first-episode schizophrenia spectrum disorders who were treated with a long-acting antipsychotic medication. There was robust improvement in core psychopathology (effect size *d* = 3.36) and functionality (*d* = 1.78), with most improvement occurring within the first six months of treatment. In contrast, improvement in subjective quality of life was less marked (*d* = 0.37) and slower, only reaching significance after 12 months of treatment. Symptom remission was achieved by 70% of patients and over half met our criteria for functional remission and good quality of life. However, only 29% met the full criteria for recovery. Patients who met the recovery criteria had better premorbid adjustment, were less likely to be of mixed ethnicity and substance use emerged as the only modifiable predictor of recovery. Only 9% of our sample achieved both functional remission and good quality of life despite not being in symptom remission. We found high rates of symptom remission, functional remission and good quality of life in patients, although relatively few achieved recovery by meeting all three of the outcome criteria. Symptom remission is not a necessary prerequisite for functional remission and good quality of life, although few non-remitters achieve other recovery criteria.

## Introduction

While the clinical course of schizophrenia is characterised by marked variability between individuals and over time, the overall outcome is poor for many patients.^1,2^ Schizophrenia was long considered a lifelong illness with little or no hope of recovery.^3^ Following the introduction of antipsychotics more than sixty years ago, treatment prospects were initially modest, with clinicians settling for outcomes such as ‘behavioural control’, ‘symptom control’, or ‘stability’.^4^ However, advances in pharmacological treatment and psychosocial interventions have heightened expectations for outcomes.^5^ Indeed, there has been a progression of treatment goals from containment, through response, to remission, and more recently, to recovery.^6^ Recovery became a focus of attention when it was recognised that symptom reduction alone was not sufficient, and that functionality and outcomes that are most meaningful to patients and families need to be considered.^3^ Outcome measures that mainly focus on symptom remission should ideally be extended to include other components of recovery, as this would better fit the needs of patients.^7^ There is growing acknowledgement that people with schizophrenia do not inevitably experience deterioration over time, and most have the potential to experience considerable symptomatic improvement and achieve a substantial degree of recovery.^8^

The lack of a widely accepted and validated definition of recovery has been an obstacle for clinicians and researchers. Recovery is a complex construct. It is multifaceted and difficult to assess, particularly across communities where psychosocial and cultural factors may influence aspects such as independent living, daily activities, education and vocational status.^4,9^ Most of the instruments developed to measure recovery were designed for use in Western cultures, and may not be appropriate for use in other settings. Shrivastava et al. highlighted the lack of consensus for defining recovery. They argue that outcome measures should be multidimensional, since social and functional improvements are not necessarily linked with antipsychotic treatment response. Importantly, they emphasise that psychosocial, vocational and functional parameters differ across communities, and propose that the decision as to which components of recovery are relevant should be taken within the cultural context.^6^

While opinions differ as to what should be included in a definition of recovery, there is general consensus that symptom remission should be one of the components.

The Remission in Schizophrenia Working Group (RSWG)^3^ laid a foundation for the measurement of remission by operationally defining a threshold for symptom severity, with no significant interference with behaviour, for a period of at least six months. These criteria are easy to apply in both clinical and research settings, and have been widely adopted. The RSWG considered remission to be a “necessary but not sufficient step toward recovery,” although they also recognised that it is not an absolute prerequisite for functional improvement. The RSWG regarded recovery as a more demanding and longer-term state than symptom remission.^3^ However, they did not provide criteria for recovery, citing the need for further evidence on the longitudinal course of recovery components. In particular, there is a need to assess relationships between symptom remission and other outcome measures, particularly functional outcome and quality of life (QOL). In the present study, we sought to address this knowledge gap by assessing the rates, trajectories, correlations and predictors of recovery across three domains (i.e. core psychopathology, clinician-rated social and occupational functioning and patient-rated QOL) in a cohort of first-episode schizophrenia spectrum disorder patients treated over 24 months. To assure treatment and circumvent nonadherence, assessments were scheduled at regular time point intervals and patients were treated with a long-acting antipsychotic medication.

## Results

We screened 234 patients for eligibility, 64 did not meet the inclusion criteria, 19 declined to participate and 18 were excluded for other reasons. Of the133 who entered the study six patients were excluded for no longer meeting the inclusion criteria and 29 were excluded for not completing 6 months of treatment. The sample therefore comprised 98 patients, of whom 72 (73%) were men and 26 (27%) women, aged 24.2 ± 6.4 years, of mixed (*n* = 75, 77%), black (*n* = 13, 13%) and white (*n* = 10, 10%) ethnicity, with a DSM-IV TR diagnosis of schizophrenia (*n* = 66, 67%), schizophreniform (*n* = 31, 32%) and schizoaffective (*n* = 1,1%) disorder. The mean duration of untreated psychosis (DUP) was 34.6 ± 44.8 weeks. The mean modal dose of flupenthixol decanoate was 11.7 ± 3.8 mg 2 weekly. Seventy-two (73%) completed 24 months of treatment. Reasons for study withdrawal were: withdrawal of consent (*n* = 10), lost to follow-up (*n* = 6), relocation (*n* = 3), incarceration (*n* = 2), poor treatment response (*n* = 2), severe side-effects (*n* = 1), general medical condition (*n* = 1), and severe drug abuse (*n* = 1). Regarding substance use, *n* = 11(11%) patients were classified as cannabis use only and n = 37 (38%) as polysubstance (cannabis, alcohol, methamphetamine and methaqualone) use.

### Improvement trajectories for the recovery domains

**Fig. 1:**
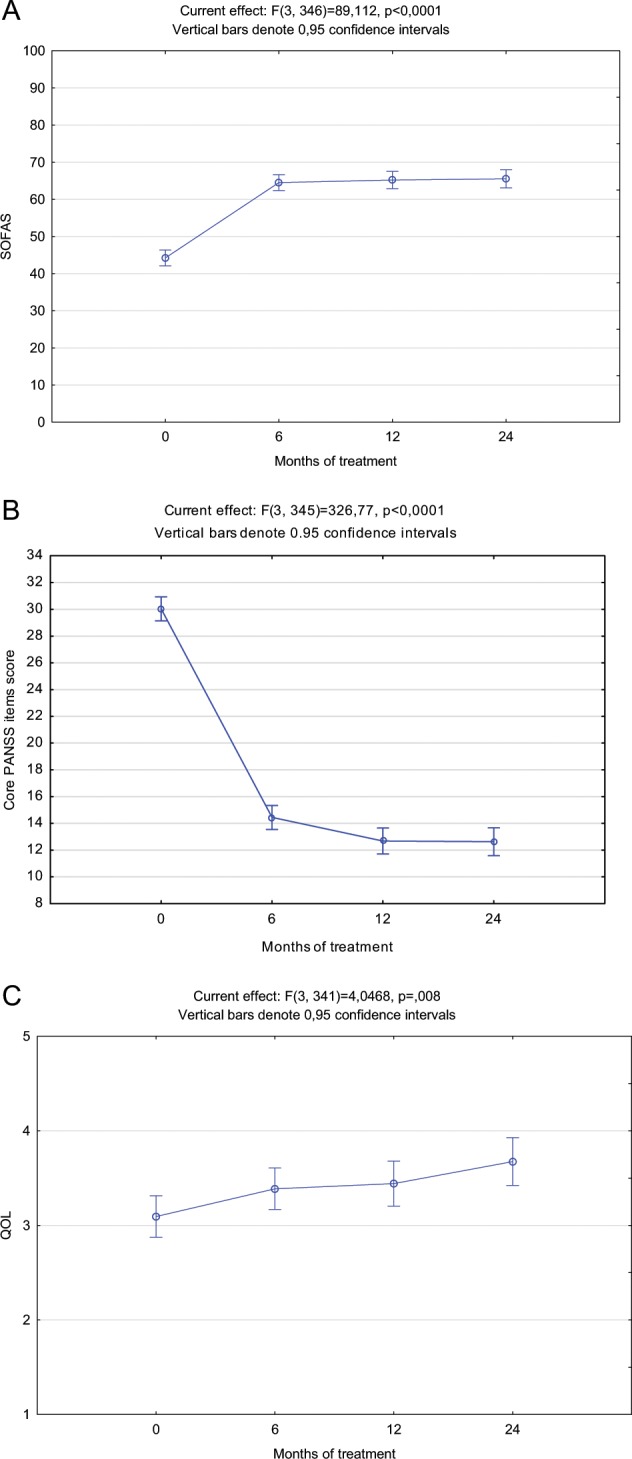
A graphical representation of the change in psychopathology, functionality and quality of life over 24 months. Mixed model repeated measures for the **a** SOFAS, **b** PANSS core items total, and **c** patient rated overall QOL scores over 24 months. SOFAS = Social and Occupational Functioning Assessment Scale, PANSS = Positive and Negative Syndrome Scale, QOL = Quality of life.

Figure 1 provides a graphical representation of the change in psychopathology (PANSS core item total score), functionality (SOFAS score) and QOL (patient-rated overall WHOQOL-Bref score) over 24 months. There were significant improvements in PANSS core item total scores from baseline to month six, and again from month six to month 12, as shown in Table 1. SOFAS scores improved significantly from baseline to month six, but not after that. Improvement in patient-rated overall QOL was slower and only reached statistical significance at month 12. None of the domains showed further significant improvement after month 12. Effect sizes for improvements from baseline to endpoint were 3.36 for PANSS core item total scores, 1.78 for SOFAS scores and 0.37 for patient-rated overall QOL.

**Table 1:** The results of the LSD tests showing changes in the three outcome domains over 24 months.

Outcome measure	Mean difference	−95% CI	+95% CI	*p*-value
Core PANSS				
Month 0 to Month 6	15.5918	14.3277	16.856	<0.001^a^
Month 6 to Month 12	1.76073	0.43995	3.08151	0.009^a^
Month 12 to Month 24	0.05210	−1.3731	1.47728	0.9
SOFAS				
Month 0 to Month 6	−20.306	−23.317	−17.295	<0.001^a^
Month 6 to Month 12	−0.693	−3.8399	2.4539	0.7
Month 12 to Month 24	−0.2977	−3.6678	3.0724	0.9
QOL				
Month 0 to Month 6	−0.2936	−0.6041	0.01688	0.06
Month 6 to Month 12	−0.3485	−0.6724	−0.0247	0.04^a^
Month 12 to Month 24	−0.2329	−0.5795	0.11362	0.2

*CI* confidence interval, *PANSS* Positive and Negative Syndrome Scale, *SOFAS* Social and Occupational Functioning Assessment Scale, *WHOQOL-BREF* World Health Organisation quality of life-BREF scale

### Recovery rates and correlations between recovery domains

**Fig. 2:**
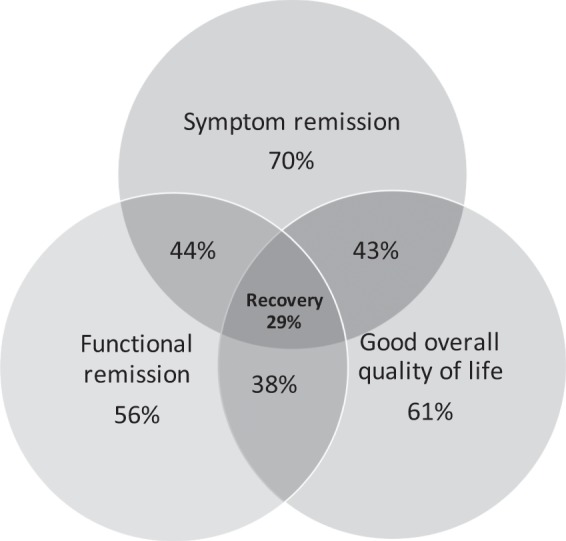
A Venn diagram illustrating the proportion of patients with individual or overlapping components of the early recovery criteria.

In Fig. 2 a Venn diagram depicts the proportion of patients meeting criteria for individual and overlapping components of the recovery criteria at endpoint. While more than half of the patients met criteria for each of the individual components, only 29% met our full criteria for recovery. Endpoint SOFAS scores were negatively correlated with PANSS core item total scores (*r* = −0.53, *p* < 0.0001) and positively correlated with patient rated overall QOL scores (*r* = 0.26, *p* = 0.01). However, the correlation between PANSS core item total scores and patient rated overall QOL scores did not reach statistical significance (*r* = −0.17, *p* = 0.1).

### Predictors of recovery

Table 2 and 3 provides a comparison of the demographic and baseline clinical scores for patients meeting recovery criteria and the rest of the sample. Those meeting recovery criteria were older, better educated, had better premorbid adjustment, were less likely to be of mixed ethnicity, less likely to use substances, and had a higher composite MCCB score. In the logistic regression analysis, substance use (OR = 9.3, 95% CI 1.6–53.0, *p* = 0.01), ethnicity, (OR 0.2, 95% CI 0.03–1.6, *p* = 0.01) and premorbid adjustment (OR = 0.003, 95% CI 0.00003–0.5, *p* < 0.001) were significant predictors of overall recovery. We then compared patients meeting criteria for the individual components of recovery to the rest of the sample. Those achieving symptom remission at endpoint had higher baseline PANSS positive domain scores (17.7 ± 3.1 vs. 16.3 ± 3.8, *t* = 2.0, *p* = 0.05) and better premorbid adjustment during late adolescence (0.3 ± 0.2 vs. 0.4 ± 0.2, *t* = −2.5, *p* = 0.02). In a logistic regression model, both baseline PANSS positive domain scores (OR 1.2, 95% CI 1.0–1.3, *p* = 0.04) and better premorbid adjustment during late adolescence (OR 0.04, 95% CI 0.003–0.5, *p* = 0.01) remained significant predictors of recovery. According to the preliminary univariate analyses patients meeting criteria for functional remission criteria were older (25.9 ± 7.5 vs 22.0 ± 4.2 yrs., *t* = 3.0, *p* = 0.004), more likely to be employed (*n* = 14/54; 26% vs. *n* = 4/43; 9%, *X*^2^ = 4.4, *p* = 0.04), less likely to use substances (*n* = 17/54; 31% vs. *n* = 30/43, 70%, *X*^2^ = 14.0, *p* = 0.0002), had lower baseline scores for the PANSS excitement/hostility domain (7.5 ± 3.7 vs. 9.1 ± 4.2, *t* = −2.1, *p* = 0.006), better general premorbid adjustment (0.4 ± 0.2 vs. 0.6 ± 0.2, *t* = −3.2, *p* = 0.002), less impairment of motor co-ordination (1.1 ± 1.3 vs. 1.7 ± 1.5, *t* = −2.1, *p* = 0.03) and better performance on the MCCB working memory domain (32.2 ± 11.9 vs. 26.4 ± 11.5, *t* = 2.2, *p* = 0.03). However, none of these variables remained significant predictors of SOFAS remission in the regression model. Finally, in the preliminary analyses patients with good or very good overall QOL at endpoint were less likely to be of mixed ancestry (*n* = 40/59; 68% vs. *n* = 34/38; 89%, *X*^2^ = 6.4, *p* = 0.04), less likely to have had a history of substance use (*n* = 23/59; 40% vs. *n* = 24/38; 63%, *X*^2^ = 5.4, *p* = 0.02), had lower baseline excitement/hostility scores (7.4 ± 3.9 vs. 9.2 ± 3.6, *t* = −2.1, *p* = 0.03), better baseline overall QOL (3.3 ± 1.1 vs. 2.8 ± 1.1, *t* = 2.1, *p* = 0.03) and better performance on the MCCB speed of processing (29.0 ± 14.0 vs. 22.6 ± 11.2, *t* = 2.1, *p* = 0.04) and reasoning/problem solving domains (39.3 ± 12.1 vs. 33.7 ± 9.6, *t* = 2.2, *p* = 0.04). However, none of these variables remained significant predictors of QOL in the regression model.

**Table 2:** Comparison of demographic profile for patients meeting recovery criteria vs. the rest of the sample.

Variables	Recovery (*n* = 28, 29%)	Rest of the sample (*n* = 70, 71%)	*t*-value	*p*-value
Age in years, mean (SD)	27.39 (7.93)	22.89 (5.34)	3.26	0.002^a^
Highest school grade passed, mean (SD)	10.67 (1.96)	9.51 (2.13)	2.44	0.017^a^
Sex, *n* (%)				
Male	20 (71%)	52 (74%)		0.80
Female	8 (29%)	18 (26%)		
Ethnicity, *n* (%)				
Africans	7 (25%)	6 (8%)		
Mixed	15 (54%)	60 (86%)		0.003^a^
White	6 (21%)	4 (6%)		
DSM-IV-TR diagnosis *n* (%)				
Schizophreniform disorder	6 (21%)	25 (36%)		0.10
Schizophrenia	21 (75%)	45 (64%)		
Schizoaffective disorder	1 (4%)	0 (0%)		
Substance use *n* (%)	5 (18%)	43 (61%)		<0.001^a^
Employment status *n* (%)				
Unemployed	21 (75%)	59 (84%)		0.20
Informal	0 (0%)	2 (3%)		
Full time	7 (25%)	9 (13%)		

*DSM-IV-TR* Diagnostic and Statistical Manual of Mental Diseases, 4th edn, Text Revision, *SD* standard deviation, *p* significance value, *T-test* for continuous variables

## Discussion

Our patients responded robustly to antipsychotic treatment in terms of psychopathology improvement, with 70% achieving symptom remission. This is consistent with previous reports of a favourable treatment response in first-episode schizophrenia,^10^ and suggests that when treatment is assured, the majority of first-episode patients will achieve sustained symptom remission. In addition to the improvements in psychopathology, over half of our participants achieved functional remission and favourable subjective QOL. Despite this, fewer than 1/3 met our recovery criteria in all three domains. While comparison of our results with those of other longitudinal studies is complicated by methodological differences, our findings are similar to others reporting poorer outcomes when domains beyond just symptom remission are considered as outcome measures.^9–12^

The improvement trajectories for the PANSS core symptom scores and SOFAS scores were similar, indicating a close relationship between these two domains. Robust improvements were observed in both domains, with most improvement occurring during the first six months of treatment and reaching a plateau at 12 months. The highly significant correlation between endpoint psychopathology and functionality provides further evidence of a close relationship between these two domains, consistent with findings reported in previous studies.^13^ However, of note is that 26% of patients achieving symptom remission did not achieve functional remission, and 12% of patients who were in functional remission did not achieve symptom remission. These findings confirm that, while symptom remission is closely related to functional remission, it is not a necessary prerequisite for functional remission.^3,6^

An association between QOL and symptom remission has been reported in previous studies.^14–16^ Patients who achieved symptom remission reported a better QOL^15^ and failure to achieve symptom remission was associated with poorer QOL.^16^ Also, a recent meta-analysis found a significant negative correlation between QOL and symptom severity.^17^

However, in our study we found that endpoint symptom remission and subjective QOL were not significantly correlated, and 27% of those achieving symptom remission did not rate their QOL as good or very good. These differences in findings may be explained by our focus on the core positive, negative and disorganised symptoms of schizophrenia, which may not be closely associated with QOL. Indeed, an earlier meta-analysis reported weak associations between psychiatric symptoms and QOL, with general psychopathology showing the strongest associations.^18^ Further, a review of studies assessing QOL in schizophrenia reported that affective symptoms were major obstacles to QOL improvement, while positive and negative symptoms were largely independent from subjective QOL.^19^ Additionally, in a pooled analysis of eight longitudinal studies, it was found that, while a reduction in psychiatric symptoms was associated with improvements in QOL, only improvements in depression/anxiety and hostility domains remained significant in the multivariate model.^20^ Finally, our findings are consistent with those reported in a recent meta-analysis conducted by Van Eck et al., which explored the relationship between clinical recovery and personal recovery, the latter being related to QOL.^7^ While the authors found a significant relationship between symptom severity and personal recovery, the association was strongest for affective symptoms and weaker for positive and negative symptoms. In our study, relative independence of QOL was further supported by the observation that the improvement trajectory in this domain was different in that it occurred more gradually, and to a much lesser degree, than symptom and functional improvement. Taken together, these findings suggest that factors other than core symptoms of the illness influence QOL, and that QOL may be less responsive to antipsychotic treatment. Interventions focusing on symptom reduction and functional improvement alone may therefore fail to improve QOL.^19^

Remission has been proposed as a necessary, but not sufficient, step toward recovery.^3^ Our results suggest that this is not necessarily the case, although few who failed to achieve symptom remission met the other recovery criteria. In fact, only 9 (9%) of our sample achieved both functional remission and good or very good subjective QOL despite not being in symptom remission.

Post-hoc *t*-test comparison of endpoint PANSS domains of these 9 patients with the rest of the sample suggests that specifically, more prominent negative symptoms account for their failure to achieve symptom remission (score of 13.3 vs 10.3, *t* = 2.03, *p* = 0.045). Although small, this might be an important subgroup to further research. Studies could explore factors other than antipsychotic treatment response contributing to their QOL and functionality. It would also be interesting to investigate the effects of both increasing and decreasing antipsychotic dose in these individuals. It could be that the antipsychotic dose is suboptimal in these patients with persistent symptoms and that increasing the dose may result in further improvements. On the other hand, it may be that these individuals are less responsive to antipsychotics and that dose reductions may benefit the patients by reducing the side-effect burden without worsening the symptoms.

Other studies have identified several significant predictors of recovery, including better educational and occupational status,^21^ shorter DUP,^10,21^ better premorbid adjustment,^22,23^ fewer negative symptoms at baseline,^24^ younger age at diagnosis,^11^ Caucasian ethnicity,^25^better cognitive functioning at stabilisation^26^ and more cerebral asymmetry.^10^ In our study, patients meeting recovery criteria were older, better educated, had better premorbid adjustment, were less likely to be of mixed ethnicity, and less likely to use substances. However, in the logistic regression analysis, only substance use, ethnicity and premorbid adjustment emerged as significant predictors of recovery. Substance use is the only modifiable predictor of recovery. Integration of substance use treatment in mental health treatment protocols and services may promote recovery in this population.

Detailed comparisons of recovery rates and predictors across studies are difficult and of limited value, given the methodological differences. Most importantly, the lack of consensus in defining and measuring recovery is problematic. Mausbach et al.^27^ reviewed eight measures of functional ability and concluded that no “gold standard” measure exists. The authors noted that most studies utilising these scales were cross-sectional, with little being known about their validity in predicting real-world health outcomes.

Similarly, a review of measures assessing QOL in schizophrenia identified 35 different scales and highlighted the lack of consensus on their clinical value.^19^ The lack of uniformly accepted criteria and assessment instruments, together with wide variation in patient populations and methodology, markedly limits generalisability of studies conducted to date. Of the recovery domains, psychopathology improvement is likely to be less influenced by environmental factors than functionality and QOL, although even expression of psychopathology may differ across cultures. For example, it has been proposed that “social kindling” could account for observed differences in auditory hallucinations across distinct cultural groups.^28^ Nevertheless, the relative stability of symptoms across populations likely contributed to the success and widespread adoption of the RSWG remission criteria,^3^ as they only consider improvements in core psychopathology. In contrast, the substantial impact of sociocultural and other environmental factors on functionality and QOL^6^ are barriers to developing broader criteria for recovery in schizophrenia. One way of minimising the impact of sociocultural differences is to use global scores for assessing functionality and QOL, which was the rationale adopted in the present study.

The strength of our study lies in the addressing of several important methodological shortcomings which defined previous studies. Using first-episode, minimally treated patients reduced the possible confounding effects of disease chronicity and previous treatment. Using standardised treatment addressed the possible confounding effects of antipsychotic heterogeneity, and treatment with a long-acting formulation removed the effect of covert non-adherence, which may be substantial in outcome studies. Finally, comprehensive characterisation of the cohort with regular assessment points allowed us to investigate changes over time in multiple outcome domains. However, there are also several important limitations that need to be considered when interpreting our findings. First, the study duration of two years, while longer than most longitudinal studies conducted in a controlled setting, does not provide an indication of the longer-term outcomes in our sample. A longer follow-up duration is particularly important when considering recovery as an outcome measure, given that the improvements should be sustained over a protracted period.

Second, our use of global measures of functionality and QOL, while helpful in circumventing the influence of environmental disparities, was not able to assess different aspects of functionality and QOL. Third, since the majority of our subjects were drawn from a socio-economically deprived community, our findings may not be generalisable to other populations. Fourth, this was a convenience sample of patients who presented to health care services and may not be representative of the larger population. Fifth, because of the small numbers of black and white participants, our findings regarding ethnicity should be treated with caution. Finally, the use of a single antipsychotic, while removing the effects of treatment heterogeneity, precludes the generalisation of our findings to patients treated with other antipsychotics.

In summary, we found high rates of symptom remission, functional remission and favourable subjective QOL in patients with a first episode of schizophrenia spectrum disorder treated over 24 months, although fewer than a third managed to achieve recovery by meeting all three of the outcome criteria. Given the need to assess treatment outcome in schizophrenia in domains beyond just symptom improvement, the development of valid, culture-free measures of components of recovery should enjoy priority amongst the research community.

## Methods

### Ethics approval

Approval to conduct the study was obtained from the Human Research Ethics Committee of Stellenbosch University Faculty of Medicine and Health Sciences. Written, informed consent was obtained from the patients and/or their legal guardians. The study registered at the South African National Clinical Trials Register (DOH-27-0710-1957; http://www.sanctr.gov.za/SAClinicalTrials/tabid/169/Default.aspx).

### Selection of study participants

This was a longitudinal single-site cohort study which included 98 first-episode schizophrenia spectrum disorder patients treated according to a standardised protocol over 24 months. Patients were recruited during their first hospital admission and at community clinics situated in a well demarcated catchment area of the eastern and northern districts of the Greater Cape Town municipality. These are multicultural areas with an estimated official unemployment rate of 23.9% and many barriers of access to mental health services. Inclusion criteria were: men and women, inpatients and outpatients, aged 16–45 years, experiencing a first psychotic episode meeting Diagnostic and Statistical Manual of Mental Diseases, Fourth Edition, Text Revision (DSM-IV-TR) diagnostic criteria for schizophrenia, schizophreniform or schizoaffective disorder. Exclusion criteria were a lifetime exposure to more than 4 weeks of antipsychotic medication, previous treatment with a long-acting depot antipsychotic, serious or unstable general medical condition and intellectual disability.

### Substance use

We included patients with substance use but excluded those who met the DSM-IV-TR criteria for substance abuse or dependence disorder. Urine toxicology screening for cannabis, methaqualone and methamphetamine was conducted at baseline and at three-monthly intervals over the 24 months of treatment.

**Table 3:** Comparison of baseline clinical scores for patients meeting recovery vs. the rest of the sample.

Variables	Recovery (*n* = 28, 29%)	Rest of the sample (*n* = 70, 71%)	*t*-value	*p*-value
DUP weeks, mean (SD)	44.59 (46.55)	30.64 (43.72)	1.40	0.17
PANSS total change at 7 weeks, mean (SD)	0.52 (0.18)	0.57 (1.93)	−0.13	0.89
PANSS, total score, mean (SD)	91.68 (18.45)	95.70 (15.05)	−1.12	0.27
PANSS, positive, mean (SD)	17.75 (3.48)	17.14 (3.32)	0.81	0.42
PANSS, negative, mean (SD)	18.79 (5.72)	20.61 (5.23)	−1.52	0.13
PANSS disorganised, mean (SD)	11.29 (2.97)	12.33 (2.72)	−1.67	0.09
PANSS excitement/hostility, mean (SD)	7.07 (3.97)	8.64 (3.76)	−1.84	0.07
CDSS total score, mean (SD)	3.75 (4.13)	3.33 (4.23)	0.45	0.66
SOFAS, mean (SD)	47.75 (14.95)	42.70 (10.43)	1.90	0.06
WHOQOL-BREF, mean (SD)				
Psychological	13.19 (2.64)	12.83 (2.53)	0.62	0.54
WHOQOL-BREF Social	13.19 (4.30)	11.43 (3.85)	1.95	0.05
WHOQOL-BREF Environment	12.15 (3.22)	11.48 (3.02)	0.96	0.34
PAS total childhood, mean (SD)	0.24 (0.17)	0.23 (0.15)	0.04	0.97
PAS total early adolescence,	0.23 (0.14)	0.32 (0.16)	−2.59	0.011^a^
PAS total late adolescence	0.27 (0.14)	0.42 (0.19)	−3.63	<0.001^a^
PAS total adult	0.35 (0.24)	0.39 (0.23)	−0.81	0.42
PAS total general	0.36 (0.14)	0.53 (0.20)	−4.15	<0.001^a^
PAS overall	0.29 (0.13)	0.38 (0.14)	−2.96	0.004^a^
NES sensory integration, mean (SD)	2.50 (2.66)	2.61 (2.09)	−0.23	0.82
NES motor coordination, mean (SD)	1.25 (1.40)	1.34 (1.46)	−0.29	0.78
NES sequencing of motor acts, mean (SD)	2.64 (2.61)	2.94 (2.35)	−0.55	0.58
NES total, mean (SD)	13.93 (8.39)	14.73 (6.84)	−0.49	0.63
BIS subscale 1, mean (SD)	1.92 (0.97)	2.40 (1.06)	−1.91	0.06
BIS subscale 2, mean (SD)	1.54 (1.28)	1.60 (1.28)	−0.20	0.84
BIS subscale 3, mean (SD)	2.36 (1.06)	2.08 (0.98)	1.15	0.25
BIS total, mean (SD)	5.82 (2.33)	6.08 (1.81)	−0.54	0.59
MCCB SoP mean, (SD)	30.88 (12.42)	24.49 (13.21)	2.00	0.049^a^
MCCB AV, mean (SD)	33.65 (11.43)	30.08 (9.75)	1.39	0.17
MCCB WM, mean (SD)	32.33 (12.48)	28.80 (11.65)	1.21	0.23
MCCB Vrbl Lrng, mean (SD)	38.38 (9.57)	34.18 (9.52)	1.80	0.08
MCCB Vis Lrng, mean (SD)	37.46 (13.80)	31.59 (13.32)	1.78	0.08
MCCB RPS, mean (SD)	41.67 (11.83)	35.09 (10.83)	2.41	0.018^a^
MCCB SC, mean (SD)	44.27 (20.55)	44.85 (20.28)	−0.11	0.91
MCCB Composite score, mean (SD)	29.17 (13.29)	22.42 (12.75)	2.06	0.043^a^

*SD* standard deviation, *MATRICS* Measurement and treatment research to improve cognition in schizophrenia, *MCCB* MATRICS consensus cognitive battery, *SOP* speed of processing, *AV* attention/vigilance, *WM* working memory, *Vrbl Lrng* verbal learning, *Vis Lrng* visual learning, *RPS* reasoning and problem solving, *SC* social cognition, *PANSS* positive and negative syndrome scale, *SOFAS* social and occupational functioning assessment scale, *CDSS* Calgary depression scale for schizophrenia, *BIS* Birchwood insight scale, *PAS* premorbid adjustment scale, *EAdol* early adolescence, *LAdol* late adolescence, *NES* neurological evaluation scale, *WHOQOL-BREF* World Health Organisation quality of life-BREF scale, *p* significance value, *T-test* for continuous variables

Alcohol use was assessed using a self-report questionnaire based on the CAGE criteria.^29^

### Antipsychotic treatment

There was a one week lead-in period of oral flupenthixol 1–3 mg per day followed by long-acting flupenthixol decanoate injections every two weeks for the duration of the study. The starting dose of flupenthixol decanoate was 10 mg two-weekly intramuscular injection (IMI), with six weekly increments of 10 mg two weekly IMI permitted, to a maximum of 30 mg two-weekly IMI. A starting dose of 5 mg 2-weekly was allowed for patients aged 18 years or younger. Additional oral flupenthixol tablets were prescribed at the discretion of the investigator for acute exacerbation of psychotic symptoms between visits. Investigators were encouraged not to increase the dose of flupenthixol decanoate too rapidly, but rather prescribe lorazepam up to 12 mg during the acute phase and thereafter up to 4 mg per day, for agitation. Prohibited medications included other antipsychotics, mood stabilisers and psychostimulants. No additional structured psychosocial interventions were routinely provided.

### Clinical assessments

Patients were assessed and diagnosis confirmed using the Structured Clinical Interview for DSM-IV (SCID).^30^

### Assessment of psychopathology, functioning and quality of life

Psychopathology was assessed using the Positive and Negative Syndrome Scale (PANSS).^31^ using the eight previously defined “core symptom” items^3^ to assess psychopathology changes over time and to define remission.

We also used factor-analysis derived domains for positive, negative, disorganised and excitement/hostility symptoms at endpoint.^32^ Depressive symptoms were assessed with the Calgary Depression Scale for Schizophrenia (CDSS).^33^ Functionality was assessed using the Social and Occupational Functioning Assessment Scale (SOFAS)^34^ derived from the Global Assessment of Functioning Scale.^35^ SOFAS estimates the overall level of social and occupational functioning at the time of assessment, and rates social and occupational functioning on a continuum from grossly impaired functioning (1–10) to superior functioning (91–100). The impairment must be a direct consequence of mental and physical health problems, but is not directly influenced by the overall severity of the individual's symptoms. The validity and reliability of this scale have been verified.^36,37^ Patient rated QOL was assessed with the World Health Organization Quality of Life-Bref (WHOQoL-Bref) questionnaire, which comprises 24 items grouped into four domains, namely physical, psychological, social and environmental, and two individual items for overall perception of QOL and satisfaction with general health. The WHOQoL-Bref is the most frequently used QoL-instrument in studies investigating patients with schizophrenia^19^ and good to excellent reliability and validity has been reported.^38^

### Recovery criteria

We defined recovery according to symptom remission, clinician rated social and occupational functioning and patient-rated overall QOL. Symptom remission was defined according to the RSWG criteria, comprising a score of mild at most on each of 8 PANSS items considered to represent core features of schizophrenia, in the positive, negative and disorganised domains. Additional requirements for symptom remission are that symptoms do not interfere significantly with functioning and are present for at least 6 months.^3^ Functional remission was defined as a SOFAS score of 61 or higher (some difficulty in social, occupational, or school functioning, but generally functioning well, has some meaningful interpersonal relationships) as previously described.^39,40^ For QOL, we used the item rating an individual’s overall perception of QOL and selected a score of 4 (good) or 5 (very good) to qualify for recovery.^7^ We chose a single item global rating for QOL to reduce effects of sociocultural factors, as well as to reduce the number of variables.

### Additional assessments

We assessed neurological soft signs with the Neurological Evaluation Scale (NES),^41^ insight with the Birchwood Insight Scale (BIS),^42^ premorbid functioning with the Premorbid Adjustment Scale (PAS),^43^ and cognitive performance with the MATRICS (Measurement and Treatment Research to Improve Cognition in Schizophrenia) Cognitive Consensus Battery (MCCB).^26^

### Rater training and reliability

Psychiatrists rated the PANSS, CDSS, ESRS and SOFAS. A comprehensive rater training by a series of videotaped and live clinical interviews was conducted prior to rating patients in the study and each rater was required to reach a score of 0.75 for the inter-rater correlation coefficient. The MCCB was carried out by research psychologists with a masters degree minimum level of qualification.

### Statistical analyses

For changes in the recovery domains over time: linear mixed-effect models for continuous repeated measures (MMRM) were constructed to assess the changes in PANSS core items total,^3^ SOFAS and WHOQOL-BREF patient-rated overall QOL scores over time, with age and sex as covariates. Within analyses, Fisher’s Least Significant Difference (LSD) post-hoc tests were used to compare the means between visits.

#### For endpoint analyses

Endpoint scores were calculated by last observation carried forward (LOCF). We only included patients who had completed at least six months of treatment, as our longitudinal evaluations indicated that the bulk of the improvements occurred during this period. Cohen’s d effect sizes were calculated for score changes from baseline to endpoint from the means and standard deviations. Pearson correlation coefficients were calculated to assess the linear relationship between the PANSS core items total, SOFAS and WHOQOL-BREF patient-rated overall QOL scores at end-point.

#### For predictor analyses

To select variables for our regression models we used T-tests for continuous variables and Chi-squared tests for categorical variables to compare those meeting recovery criteria with the rest of the group. Differences at the *p* = 0.1 significance level were used to select predictor variables for logistic regression with recovery (yes/no) as the dependent variable.

We constructed similar logistic regression models for dependent variables of PANSS remission status, SOFAS remission status and WHOQOL-BREF patient-rated QOL.

### Reporting summary

Further information on research design is available in the Nature Research Reporting Summary linked to this article.

## Supplementary information

reporting summary file

## Data Availability

The data that support the findings of this study are available from the corresponding author upon reasonable request.
